# Reunion of Charing Cross Hospital Students

**Published:** 1911-10-07

**Authors:** 


### Reunion of Charing Cross Hospital Students.

The annual dinner of past and present students of Charing Cross Hospital was held on Monday evening, October 2, in the new Adelaide Gallery just opposite the Hospital. The chair was taken by Dr. Cuthbert Lockyer, Lecturer on Practical Obstetrics at Charing Cross, who was supported by Mr. Alban Doran, Consulting Surgeon to the Samaritan Hospital. The toast of the Hospital and School was proposed by Dr. Lockyer, who, an old Charing Cross man himself, recalled many interesting reminiscences. The changes in the teaching arrangements at the school were naturally made the subject of many references by the Dean, Dr. W. Hunter, and subsequent speakers, the hopeful tone and earnest spirit of whose speeches augurs well for a successful issue from a somewhat chequered past. Dr. Mott, in a happy vein, coupled the toast of "The Visitors" with the name of Mr. Alban Doran, Dr. McPhail, Lecturer on Anatomy, and Mr. Clifford Smith, Consulting Engineer to the London County Council. A pleasant interruption was made for the presentation of a testimonial to the senior porter, who was retiring after serving the hospital for thirty-five years. Mr. Alban Doran's speech showed that he had been on intimate terms with the leading lights of Charing Cross Hospital in the days gone by. Mr. James Cantlie made a characteristic speech, which added much to the gaiety of the evening. The Chairman's health was proposed by Dr. Amand Routh, Obstetric Physician to the Hospital. The gathering this year was specially successful, and the attendance was a good one.

During the dinner the following autograph letter was received from Princess Louise :—

Kensington Palace, W.
2nd October, 1911.

Dear Dr. Hunter,—I would like you to convey from me, as President of the Charing Cross Hospital, my congratulations to the students on the reorganisation of their school.

These improvements will in every way give them greater facility and comfort to carry on their studies and research thoroughly.

The zeal and enthusiasm shown by their Dean and his colleagues in the reorganisation of the College, in remodelling it and bringing it to the present state of efficiency, will, I am sure, stimulate the students to an even keener desire for success than has to-day been evinced, and a wish to send out the ablest men who will leave their mark in the world in the future, bringing lasting fame to the College.

Let me again thank you, as Dean, and also all your colleagues for having brought these schools to their present high standard.

If only the public would realise what great work is being carried on and what uphill work it is to keep up the efficiency with inadequate means, and were to reflect that it is all for the benefit of the general public, they would be ready to help.

The building expenses are now over, and it is the upkeep, as the Duke said, which is needing help.

I wish you the realisation of all your hopes.

Believe me, yours sincerely,
(Signed) Louise.

